# Drug screen identifies verteporfin as a regulator of lipid metabolism in macrophage foam cells

**DOI:** 10.1038/s41598-023-46467-4

**Published:** 2023-11-09

**Authors:** Nicholas Hoeffner, Antoni Paul, Young-Hwa Goo

**Affiliations:** Molecular and Cellular Physiology Department, Albany Medical College, Albany, NY 12208 USA

**Keywords:** Cardiology, Diseases

## Abstract

Arterial macrophage foam cells are filled with cholesterol ester (CE) stored in cytosolic lipid droplets (LDs). Foam cells are central players in progression of atherosclerosis as regulators of lipid metabolism and inflammation, two major driving forces of atherosclerosis development. Thus, foam cells are considered plausible targets for intervention in atherosclerosis. However, a compound that directly regulates the lipid metabolism of LDs in the arterial foam cells has not yet been identified. In this study, we screened compounds that inhibit macrophage foam cell formation using a library of 2697 FDA-approved drugs. From the foam cells generated via loading of human oxidized low-density lipoprotein (oxLDL), we found 21 and 6 compounds that reduced and enhanced accumulations of lipids respectively. Among them, verteporfin most significantly reduced oxLDL-induced foam cell formation whereas it did not display a significant impact on foam cell formation induced by fatty acid. Mechanistically our data demonstrate that verteporfin acts via inhibition of oxLDL association with macrophages, reducing accumulation of CE. Interestingly, while other drugs that reduced foam cell formation did not have impact on pre-existing foam cells, verteporfin treatment significantly reduced their total lipids, CE, and pro-inflammatory gene expression. Together, our study identifies verteporfin as a novel regulator of foam cell lipid metabolism and inflammation and a potential compound for intervention in atherosclerosis.

## Introduction

Atherosclerosis is a chronic inflammatory disease and an underlying condition of many metabolic diseases. Endothelial dysfunction followed by increasing infiltration and subendothelial retention of apolipoprotein B containing lipoproteins (apoB-Lps) initiates plaque buildup in arteries. In atherosclerotic plaques, low-density lipoproteins (LDLs), the major sterols (e.g. cholesterol) carrying apoB-Lps, are abundantly found in their oxidized forms, and oxLDLs are continuously endocytosed by macrophages via scavenger receptors (*CD36* and *SR-A*), which are responsible for 70 ~ 80% of LDL uptake in macrophages^1–3^. Lipids carried by oxLDL are then hydrolyzed by the lysosomal acid lipase (*LAL*) in the late endosome^4^. Metabolized lipids are ligands of nuclear receptors that directly regulate the expressions of genes involved in lipid metabolism. Peroxisome proliferator-activated receptor γ (*PPAR*γ) induces cluster of differentiation 36 (*CD36*) expression in oxLDL-treated macrophages^5,6^. Free sterols such as free cholesterol (FC) and oxidized free sterols (oxysterols) are ligands of liver X receptor (*LXR*) which transcriptionally induces the expressions of plasma membrane transporters, ATP-binding membrane cassette transporter A1 and G1 (*ABCA1* and *ABCG1)* to efflux FC to extracellular receptors and to antagonize inflammatory signaling cascades^7–12^. When the efflux pathway is saturated, free sterols are re-esterified with fatty acids (FAs) in the ER by acyl-coenzyme A: cholesterol acyltransferases (*ACAT*), and the esterified sterols are immediately surrounded by LD-associated proteins, forming lipid droplets (LDs) to be released to cytosol and resulting in foamy appearance of macrophages so-called foam cells^13,14^. The LD is a multifaceted organelle that regulates cellular metabolism. It regulates lipid metabolism from uptake, distribution, storage, and efflux and is involved in multiple signaling pathways by sequestering and releasing lipid-mediators in a timely manner. The esterification of cytotoxic FAs and FCs into inert neutral lipids protects cells from lipid-induced cell death^15,16^. However, overwhelming endocytosis of oxLDLs by macrophages followed by the dysregulation of lipid metabolism and cell death leads to the burst of lipid mediators and defective efferocytosis, contributing to the formation of necrotic core in plaques, leading to plaque instability and rupture^17^.

Given that macrophage/foam cells are major players in atherogenesis, targeting these cells to intervene in atherosclerosis has been an object of aspiration. For example, knock out (KO) of *Plin2*, a LD associated protein, in macrophages reduced foam cell formation and prevented atherosclerosis development in apolipoprotein E knock out mice (*Apoe* KO)^18^. Also, combinatory strategy that enhances cholesterol efflux by increasing the level of human APOA1 in the circulation and prevents LD formation by *Plin2* KO significantly reduced the growth of plaques while increasing their stability^19^. Thus, targeting foam cells seems beneficial to intervening in atherosclerosis development. Administration of LXR agonists to LDL receptor KO (*Ldlr* KO) and *Apoe* KO mice prevented plaque growth via stimulation of reverse cholesterol transport and reduction of inflammatory gene expressions^20^. However, systemic administration of synthetic LXR agonist induced expression of lipogenic genes as a side effect^21^.

In clinics, therapeutic efforts have been made in lowering blood cholesterol level or inflammation systemically. Over the last several decades, pharmaceutical intervention of atherosclerosis has been carried out by statins which are presently the primary means by which hypercholesterolemia is treated in clinics^22,23^. These drugs block HMG-CoA reductase, the enzyme catalyzing the rate limiting step of de-novo cholesterol synthesis pathway. This inhibition leads to an increase in expression of LDL receptors, allowing for LDL to be removed from the bloodstream more efficiently by other tissues such as the liver^24^. While statin therapy has done much to better the prognosis of atherosclerosis patients, atherosclerosis’s status as the greatest cause of mortality in the world remains unchanged. Recently, inhibitors of proprotein convertase subtilisin/kexin type 9 (PCSK9) which increases LDLR lifespan, resulting in significant reduction of circulating LDL cholesterols, are being used in the clinic^25^. While these drugs lower circulating LDL cholesterol level systemically, compounds that directly reduce lipid burden in macrophage/foam cells are embedded in arterial walls remain unidentified. A new approach that could be used in conjunction with or in place of statins or PCSK9 would do much to improve clinical outcomes.

In this study, we aimed to identify compounds that directly regulate lipid metabolism of oxidized sterol rich macrophage/foam cells. Drug repurposing has been widely performed in search of new compounds due to its merit of acceleration of clinical application^26^. Thus, we used an FDA-approved drug library that contains 2697 compounds. Macrophage foam cells were generated by highly oxidized LDL loading to mimic oxidized cholesterol-rich environment of plaques^10^. Our study found that verteporfin has the most significant effect on the lipid content and inflammation of the oxLDL-induced macrophage foam cells. Interestingly, we also found verteporfin preferentially acts on cholesterol ester (CE)-rich LDs rather than triacylglycerol (TAG)-rich LDs. Our study presents the identification of a potential compound that directly regulates lipid metabolism of macrophage foam cells and may be applicable in establishing a new therapeutic strategy for atherosclerosis intervention.

## Results

### Drug screening displayed that over 100 compounds regulate LD formation in macrophage foam cells

**Figure 1:**
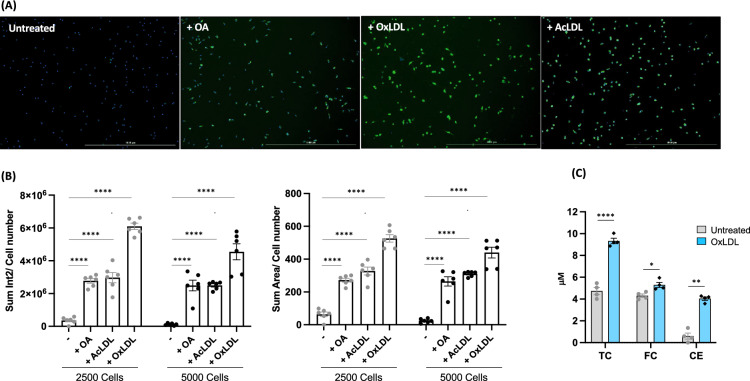
Establishment of foam cell detection (**A**) Images of foam cells treated with oleic acid (OA), oxidized LDL(OxLDL) and acetylated LDL(AcLDL) and stained by BODIPY 493/503, scale bar 1000μm. (**B**) Sum intensity normalized by cell number (left) and sum area normalized by cell number (right). Six areas containing an average 140 cells of each treatment were analyzed. (**C**) Total, free, and esterified cholesterols normalized by cell numbers. TC-total cholesterol, FC-free cholesterol, and CE-cholesterol ester. *P* values are by Student’s *t* test for paired samples (treated vs. untreated), ****P < 0.00005, **p < 0.005, and *p < 0.05.

**Figure 2:**
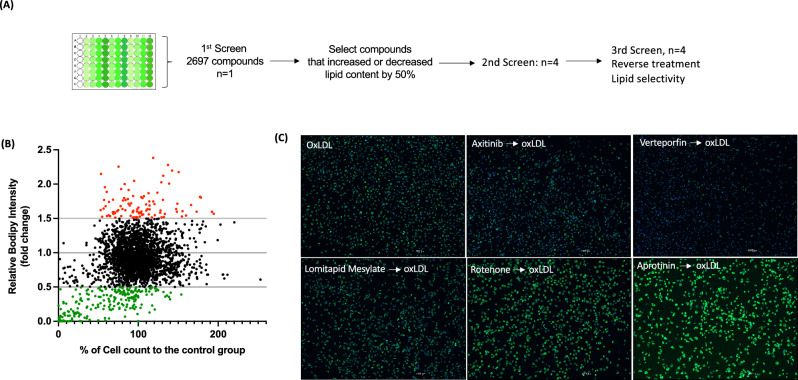
Identification of drugs that impact foam cell formation (**A**) Experimental strategy. (**B**) Distribution of BODIPY value of all compounds (2697). Relative intensity of a compound treated to oxLDL-treated group. Red dot indicates 50% higher and green dot indicates 50% lower than oxLDL-treated group. (**C**) Images of cells treated with oxLDL (control) and pretreated with indicated drugs followed by oxLDL loading. Scale bar 1000μm.

To establish a large-scale foam cell detection system, we treated mouse bone marrow derived macrophages (BMMs) in a 96 well plate with oleic acid (OA), acetylated LDL (acLDL), or highly oxidized LDL (oxLDL) to generate cytosolic LDs (Fig. 1A). LDs and nuclei were stained with BODIPY 493/503 and DAPI, respectively. The total area and intensity of LDs in each image were measured and divided by cell number. In all lipids treated cells, the intensity, and the area of LDs in each cell were increased by lipid loading and showed almost identical patterns within the same treatments. In addition, these parameters were consistent regardless of the cell density (Fig. 1B). Thus, we initiated a compound screening to identify a drug(s) regulating foam cell formation based on the screen system we have established. For the choice of lipids, we chose oxLDL because oxidation of LDL is abundantly found in and induces foam cell formation in the atherosclerotic plaques while OA and acLDL are typically favored to study LD biology. Moreover, the levels of oxysterols are increasing in the foam cells of atherosclerotic plaques as atherosclerosis progresses and are positively related to plaque vulnerability^27–29^. Moreover, in our previous study, we found enriched oxidized lipid species in BMMs treated with human oxLDL that are similar to ones found in human plaques^10^. Therefore, in the present study, we treated BMMs with highly oxidized LDL (oxLDL) to mimic in vivo foam cells largely enriched with oxidized lipids^10^. Biochemical measurement of total cholesterol (TC) and free cholesterol (FC) showed dose dependent increase of LD formations as shown in increasing levels of cholesterol ester (CE) with oxLDL loading, ensuing our foam cell detection system is suitable for assessing LDs (Fig. 1C). For the first screening we treated BMMs with 2697 FDA-approved compounds prior to oxLDL loading (Fig. 2A). For each compound, mean cellular lipid intensity was calculated and presented as fold change relative to cells treated with oxLDL only (Fig. 2B). We found that a total of 99 compounds increased, and 219 compounds decreased lipid intensity by 50% compared to the control groups treated with oxLDL alone (Fig. 2B and C). We also assessed the cytotoxicity of each compound by comparing the cell number in each image to those of control groups. Among those 318 drugs with a strong impact on LD intensity, we eliminated those that significantly lowered cell counts and chose top 100 drugs that displayed more 50% reduction and induction of lipid content to pursue for a second round of screening.

### Verteporfin inhibits foam cell formation via blocking LDL binding to macrophages

**Figure 3:**
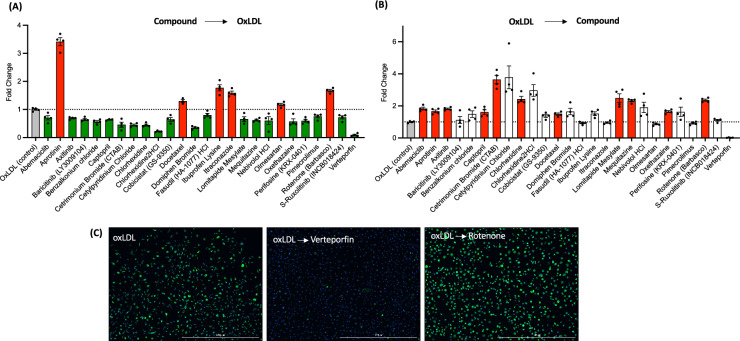
Second screening was performed with the 100 compounds selected from 1st screen. Fold change of lipid contents to control (oxLDL treated) group. (**A**) Compounds were treated prior to oxLDL loading. Twenty-six drugs that significantly decreased (green bar) and increased (red bar) foam cell formation are presented. *P* values of all colored groups (red and green bars) are < 0.05 by Student’s *t* test for compared samples (control vs compound treated). (**B**) The effect of compounds on lipid contents of 26 compounds from (**A**) were measured after oxLDL loaded for 24 h. *P* values of all colored groups (red and green bars) are below 0.05. N = 4 per group. (**C**) The representative images of post drug treatment. Scale bar 1000μm.

**Figure 4:**
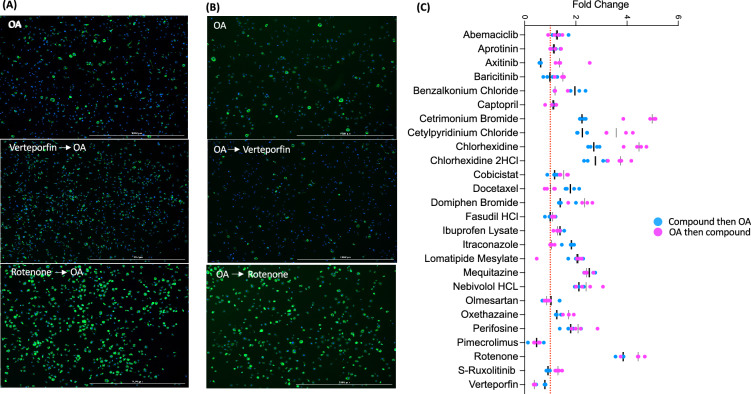
Verteporfin reduces the formation and removal of OA treated foam cells but less impactable than oxLDL treated foam cells (**A**) Representative images of foam cells pretreated with drugs and then stimulated with OA. (**B**) Representative images of foam cells that treated with OA first then with drugs. Scale bar; 1000μM. (**C**) Comparison of the impact of 26 drugs treatments before or after OA loading, n = 4 per group. Fold change is relative BODIPY intensity of OA treated group.

**Figure 5:**
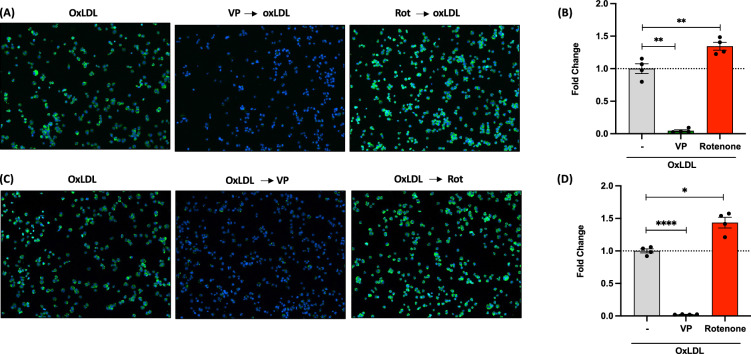
Verteporfin decreases lipid contents in human macrophage THP-1 cells. (**A**) Images and (**B**) relative BODIPY intensity of VP (10 μM) and Rotenone (10 μM) pre-treated foam cells. (**C**) Images and (**D**) relative BODIPY intensity of foam cells treated with VP and Rotenone. *P* values are by Student’s *t* test for paired samples. *p < 0.05, **p < 0.005, ****p < 0.0005.

**Figure 6:**
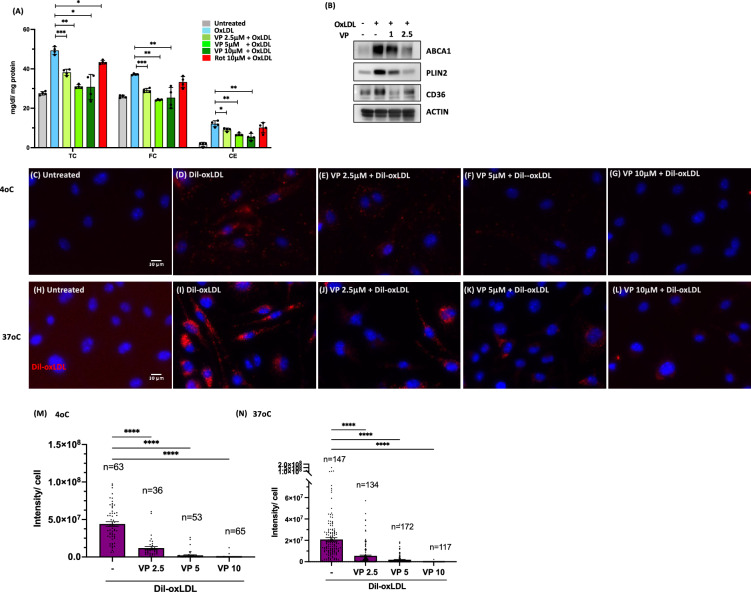
Verteporfin (VP) interferes with LDL association with macrophages. (**A**) BMMs were pretreated with drugs for 30 min before treating with oxLDL for 24 h. Total cholesterol (TC) and free cholesterol (FC) were measured as described in methods. Cholesterol ester (CE) was obtained by subtracting FC values from TC. N = 4 per group. *P* vales are by Student’s *t* test for compared groups (oxLDL treated vs. VP treated with oxLDL) (**B**) Expression levels of CD36, PLIN2 and ABCA1 in VP pretreated (1 and 2.5μM) foam cells (immunoblotting). (**C**–**G**) VP was pretreated at 0, 2.5, 5, and 10μM followed by Dil-oxLDL (10 μg/ml) for 2 h at 4 oC. (**H**–**L**) Decreased internalized oxLDL by VP preincubation. VP was pretreated at 0, 2.5, 5, and 10μM followed by Dil-oxLDL (10 μg/ml) for 3 h at 37 °C. (**M**) and (**N**) Quantification of fluorescence intensity on images of **E**–**I** and **G**–**N**, respectively. *P* vales are by Student’s *t* test for compared groups (with and without VP treatment) (****P < 0.00005, ***P < 0.0005, **p < 0.005 and *p < 0.05).

For the second screening, we increased the numbers (n = 4) of each treatment. We found that 20 and 6 compounds significantly reduced and increased foam cell formation respectively, in a manner consistent with the first screening results (Fig. 3A). The most significant reduction of foam cell formation was found in verteporfin treated groups whereas aprotinin treated cells displayed the highest LD contents (Fig. 3A). For the compounds that consistently either increased or decreased foam cell formation from the 1st and 2nd screenings, we tested whether these compounds could regulate lipid contents of pre-existing foam cells by adding drugs 24 h after oxLDL loading. Interestingly, most drugs that reduced foam cell formation had no effect or increased LD content when treated with oxLDL first (Fig. 3B). However, some drugs showed the same reduction or induction of foam cell formation regardless of the order of drug treatment and lipid loading. Verteporfin again most significantly reduced LD contents, and rotenone, ibuprofen lysate, and aprotinin increased lipid contents (Fig. 3B and C). The major esterified neutral lipids in the LDs of macrophage foam cells are CE (75%) whereas TAG is the major component of LDs of liver and adipose tissues^10,30^. Therefore, specific regulation of CE metabolism in macrophage foam cells is important to avoid side effect of lipid accumulation in other tissues^21,31^. To determine whether 26 compounds that displayed an impact on oxLDL-induced foam cells show selective regulation toward CE- or TAG-rich LDs, we measured their impacts on TAG-rich foam cells. BMMs were added with OA before or after the treatments of 26 compounds, Interestingly, most drugs increased LD contents regardless of OA loading order, but some drugs have clear preference toward lipid substrates. Some drugs that lowered oxLDL-induced LDs increased OA-induced LDs. Verteporfin and pimecrolimus reduced LD content in OA-treated BMMs regardless of their treatment order with respect to OA, but the effect was less than it was on oxLDL-induced LDs (Fig. 4A–C). Intriguingly, only rotenone still elevated OA-mediated TAG formation wherein aprotinin did not have impact on OA loaded LDs in contrast to its activity of increasing LD content on oxLDL-loaded foam cells shown in Fig. 3. Overall, verteporfin consistently reduced lipid contents in oxLDL-induced foam cells. To test whether verteporfin regulates lipid metabolism in human macrophages, THP-1 macrophages were treated with verteporfin and rotenone before and after oxLDL loading. Verteporfin and rotenone decreased and increased LD accumulation respectively (Fig. 5), confirming these drugs regulate lipid content of both human and mouse foam cells. In atherosclerotic plaque, aggregated LDLs (agLDLs) are also abundantly found, and they are readily taken up by macrophages in contrast to native LDLs (LDLs)^32,33^. AgLDL induces LD formation while it does not contain oxidized sterols such as 7-ketocholesterol that are abundant in atherosclerotic plaques^32,34^. To determine whether verteporfin also interferes with agLDL mediated foam cell formation and regression, we treated BMMs with agLDL. Pre-treatment and post-treatment of verteporfin on agLDL treated BMMs also reduced lipid contents assessed by Bodipy intensity (Supplementary Fig. 1). To investigate whether the reduced foam cell formation caused by verteporfin is reflected in the cellular cholesterol contents, we measured cellular cholesterols of BMMs loaded with oxLDL after treatments of verteporfin. Verteporfin treatment significantly reduced total levels of cholesterol and CE in a dose dependent manner (Fig. 6A). The net levels of cholesterol in cells are determined at multiple stages of cholesterol trafficking including LDL uptake, cholesterol efflux to extracellular acceptors, LD synthesis, and CE hydrolysis at LDs. Expression of genes involved in lipid trafficking are elevated when macrophages were loaded with oxLDL^3,5,6,35^. As reported, we observed oxLDL treatments increased the expression of *ABCA1*, *CD36*, and *PLIN2* in oxLDL-treated BMMs (Fig. 6B). However, pre-treatment with verteporfin reduced strength of this induction (Fig. 6B). To demonstrate the decreased CE accumulation accompanied by reduced expression of *ABCA1*, *PLIN2*, and *CD36* is due to interference of LDL uptake by verteporfin, we performed LDL uptake assay. BMMs were treated with the 3,3-dioctadecylinodocarbocyanine iodide labeled oxLDL (dil-oxLDL) following the verteporfin pretreatment^36^. The binding (Fig. 6C–G, M) and internalization (Fig. 6H–L, 6N) of dil-oxLDL in macrophages were both significantly decreased in verteporfin-treated cells in a dose dependent manner, suggesting that verteporfin interferes with LDL binding to macrophages resulting in less LD formation and lipid accumulation.

### Verteporfin treatment on pre-existing foam cells reduced lipid content and decreased inflammatory gene expression

**Figure 7:**
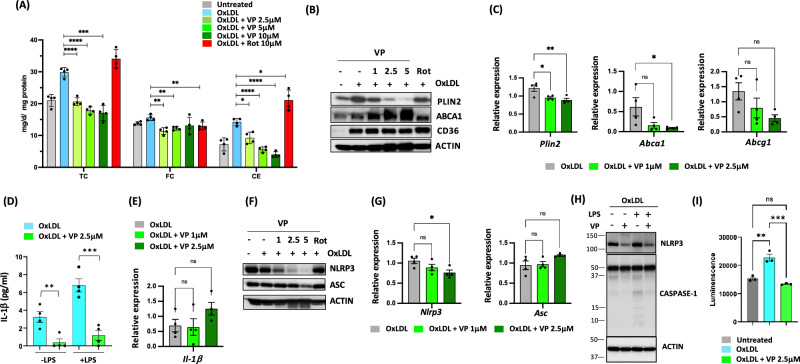
Treatment of verteporfin to pre-existing foam cells reduces CE contents and inflammation. (**A**) Foam cells were induced with oxLDL then washed and treated with drugs (μM) as indicated. Total cholesterol (TC) and free cholesterol (FC) were measured as described in methods. Cholesterol ester (CE) was obtained by subtracting FC values from TC. N = 4 per group. (**B**) Protein levels of CD36, and PLIN2 in VP pretreated (μM) foam cells (immunoblotting) and (**C**) mRNA expression of PLIN2, ABCA1, and ABCG1 relative to cyclophilin (qPCR). (**D**) IL-1β secretion and (**E**) expression (qPCR), N = 4 per group. (**F**) Protein levels of NLRP3 and ASC in VP pretreated (μM) foam cells (immunoblotting) and (**G**) mRNA expression of NLRP3 and ASC relative to cyclophilin (qPCR). (**H**) Immunoblot of cleaved capase-1 and (**I**) detection of caspase-1 activity in cultured supernatant. *P* vales are by Student’s *t* test for compared groups (with and without VP treatment) *0.005 < p ≤ 0.05, **0.0005 < p ≤ 0.005, ***0.00005 < p ≤ 0.0005, ****P < 0.00005.

In Fig. 3, we showed that verteporfin not only blocked foam cell formation but also converted foam cells into non-foamy cells without inducing cell apoptosis (Supplementary Fig. 2). In line with this observed phenotypic regression of LDs, direct measurement of cholesterol levels showed that CE stores were also significantly reduced when verteporfin was administered to pre-existing foam cells. Likewise, rotenone increased CE accumulation in pre-existing foam cells (Fig. 7A). In addition, the immunoblot showed the reduced PLIN2 protein level, reflecting our observed reduction of LDs (Fig. 7B). It is reported that mRNA of ABCA1 increases during foam cell formation and reduces during foam cell regression^37^. We also observed the reduced mRNA of ABCA1 and PLIN2 in the foam cells treated with verteporfin (Fig. 7C), indicating regression of foam cells. Interestingly we observed the protein levels of ABCA1 is increased when verteporfin was added (Fig. 7B). It is known that ABCA1 protein is stabilized when translocalized to plasma membrane for cholesterol transport, and it also plays an inhibitory role in inflammatory signaling. Lipid metabolism and inflammation are closely connected, and the inflammatory status of foam cells is an important player in regulation of plaque stability. To investigate whether reduced lipid content impacts the inflammatory status of foam cells, we assessed the secretion of a key pro-inflammatory cytokine, IL-1β. In oxLDL-induced foam cells with or without LPS priming, verteporfin treatment reduced IL-1β secretion (Fig. 7D) without affecting mRNA expression (Fig. 7E). The expression, processing, and secretion of IL-1β is controlled by NOD-, LRR, and pyrin domain-containing protein 3 (NLRP3) inflammasomes-mediated activation of capase-1, which is required for processing of pro-IL-1β and secretion^38,39^. NLRP3 inflammasomes components are enriched in inflamed human atheroma, and it is known that the inhibition of NLRP3 inflammasome enhances plaque stability^40,41^. Immunoblot of NLRP3 components, NLRP3 and ASC, in verteporfin treated cells showed significant decrease of NLRP3 expression in both levels of protein and RNA, indicating the possible defect of NLRP3 inflammasome function (Fig. 7F and G). Supporting this, we found that cleaved caspase-1 was inhibited by verteporfin treatment, and elevated activity of capase-1 in foam cells were significantly reduced by verteporfin (Fig. 7H and I). Thus, verteporfin treatment interferes with NLRP3 inflammasome function causing the defect of pro-caspase-1 maturation followed by reduced pro-IL1β processing and decreased secretion of IL1β. Together, the results suggest that the beneficial effects of verteporfin on macrophages/foam cells are the result of regulation of lipid metabolism and inflammation.

## Discussion

Macrophages/foam cells are major players in the progression of atherosclerosis due to their role in regulating lipid metabolism and inflammation from plaque initiation and expansion to rupture. Therefore, macrophages/foam cells are considered a potential target to treat atherosclerosis. While inhibiting LDL uptake seems to be a plausible strategy to reducing foam cell formation and thus blocking atherosclerosis development, it is important to consider that LDL infiltrated to the intima needs to be cleared from the lesion. Intimal LDLs in the extracellular space contribute to the formation of cholesterol crystals, which stimulate inflammation of macrophages/foam cells. The major pathway of LDL clearance is through phagocytosis by macrophages followed by metabolism of lipids within the cells and/or exporting lipids to extra-cellular receptors. However, excessive endocytosis of oxLDL by macrophages induces lysosomal rupture that activates formation of NLRP3 inflammasome, which is required for maturation and secretion of pro-inflammatory cytokines. Addressing the lipid burden in already formed foam cells in conjunction with lowering plasma cholesterol is therefore desirable. The optimal strategy would thus be concurrent treatments targeting these two aspects of lipid accumulation.

In this study, we aimed to find a drug(s) that directly regulates the lipid metabolism of macrophage foam cells by using an FDA-approved drug library. We found that verteporfin reduces lipid content in oxLDL-induced foam cells. Our results also show that verteporfin inhibits oxLDL association with macrophages and the treatment of pre-existing foam cells with verteporfin also reduces lipid levels and expression of key inflammatory genes and cytokine secretion by disrupting NLRP3 inflammasome function. The significance of this study is twofold. First, it suggests the possibility of drug repurposing to treat atherosclerosis. Second, our data on the effects of FDA-approved drugs on foam cell formation might prove useful in evaluating whether some drugs currently used for long-term treatment might inhibit or exacerbate coronary atherosclerotic disease as a side effect. However, the limit of the current study is the lack of in vivo studies assessing whether verteporfin treatment or other drugs identified can prevent the progression or regress of atherosclerotic plaque development as well as the status of atheroma inflammation in vivo.

Verteporfin is clinically used for treating choroidal neovascularization caused by age-related macular degeneration as a means of generating radicals in a light-dependent manner^42^. Thus, it is plausible to treat atherosclerosis mouse models with verteporfin to evaluate its function in the development of atherosclerosis. Interestingly, recent reports support the beneficial effects of verteporfin in lipid-related metabolic diseases. Verteporfin has been shown to disrupt the interaction between Yes-associated protein (YAP) and transcriptional coactivator with PDZ-binding motif (TAZ) independent of light exposure and prevent outgrowth of the oncogenic liver^43^. Inhibition of YAP-TAZ activity using morpholino oligos in *ApoE* mice attenuated their atherogenic phenotype and lesion growth^44^. In a recent report, overexpression of YAP and knock out of YAP in myeloid cells of *ApoE* KO mice increased and decreased atherosclerosis progression respectively, suggesting YAP as a therapeutic target of atherosclerosis^45^. Additionally, YAP activates NLRP3 inflammasome by blocking the degradation of NLRP3^46^. Thus, our finding of verteporfin, a YAP/TAZ inhibitor, as an inhibitor of foam cell formation and inflammation points to verteporfin as a plausible compound to treat atherosclerosis patients and is consistent with the current literature. Moreover, the finding that verteporfin significantly reduces the lipid content of the pre-existing foam cells indicates its use for not only preventing atherosclerosis but also treating it. Further investigation will be required to fully evaluate whether verteporfin and its analogs might be applied to the treatment of atherosclerosis, but our study provides some evidence that this repurposing could be beneficial.

Future in vivo atherosclerosis studies should provide a better picture of this drug as a potential atherosclerosis treatment. Further effectiveness could be achieved via a drug delivery system specially targeting lesional macrophages.

## Methods

### Reagents

Oxidized LDL-highly oxidized LDL, Dil-hi-oxLDL, and native LDL (nLDL) were purchased from Kalen Biochemical. Aggregated LDLs (agLDL) were prepared by vertexing nLDL as previously described^32^. The FDA approved drug library is from Selleckchem, and all other chemicals are from Sigma. The anti-ABCA1, beta Tubulin, CD-36, and PLIN2 antibodies were obtained from Novus Biologicals. The anti-ASC antibody was from Cell Signaling, and the anti-NLRP3 and Caspas-1 antibodies were purchased from Adipogen.

### Cell culture

To harvest bone marrow macrophages (BMMs), wild type mice (8 ~ 10-week-old mice) were sacrificed using isoflurane according to the protocol approved by Albany Medical Center IACUC. Femurs and tibias were removed from the mice and the bone marrow was flushed with RPMI 1640 media supplemented with 1x Antibiotic-Antimycotic. The resulting monocytes were cultured in RPMI 1640 media supplemented with 10% FBS, 1x Antibiotic-Antimycotic, and 15% L929 conditioned media to stimulate differentiation into macrophages.

### Lipid droplet measurement

Fully differentiated BMM were plated in a black/clear bottom 96 plate at density of 12,000 ~ 15,000 cell per well. Cells were treated with each compound at 10μM for 30 min followed by treatment of oxLDL (25μg/ml) for 24 h. Eight wells on each plate were left untreated or treated with oxLDL only to serve as negative and positive controls respectively. For the 2nd screening, n = 4 replicates per treatment were included. After washing cells with 1xPBS, the cells were fixed with 4% p-formaldehyde and stained with DAPI to stain nuclei and facilitate cell counting as well as BODIPY 493/503 to stain lipids and allow for calculation of lipid accumulation. Fluorescent images were taken on 4 × magnification of a 1973μm × 1457μm area in each well using the Cytation 5 (BioTek) with excitation and emission wavelengths of 377 and 447 respectively for DAPI and 469 and 525 respectively for BODIPY. ‘Autoexpose’ was used to find an ideal camera gain in the BODIPY channel for each positive control well. The median of the 8 camera gain values was then chosen to be used for the imaging of all wells on the plate. The exposure time for each plate was adjusted by eye and the LED intensity was kept constant at 10. Images were analyzed using preprocessing and cellular analysis in Gen5 v3.08. Images were subjected to preprocessing using a rolling ball diameter of 200 μm. Nuclei were identified as objects in the DAPI channel between 5 and 30 μm in diameter with an intensity greater than 5000. Areas of BODIPY intensity greater than 10,000 surrounding the nuclei were subject to analysis of BODIPY intensity. The number of cells and the total BODIPY intensity of all cells in the image were calculated and the BODIPY intensity of each image was divided by its cell count to find the BODIPY intensity/cell which we have taken as a proxy for lipid droplet buildup. The fold change in BODIPY intensity/cell brought about by each drug was calculated relative to the mean of the positive controls. We then screened for compounds that brought about a large fold change in either direction without inducing appreciable cytotoxicity.

### Real-time qPCR

Total RNA of cells was extracted with TRIZOL and purified with Agilent RNA purification Kit. cDNA was synthesized with Superscript II cDNA synthesis kits using oligo-dT primer. Real-time qPCR was performed with SYBR green mixture (Agilent) on Agilent Mx3000. Relative expression was calculated by using 2^ΔΔCt and normalizing to the level of cyclophilin.

### Dil-oxLDL uptake assays

BMMs were grown to confluence in a 96-well plate in RPMI 1640 media with 10% FBS, 1x Antibiotic and Antimycotic, and 15% L929 conditioned media. Cells were treated with varying concentrations of verteporfin for 30 min before treatment with 10ug/mL DiI-hi-oxLDL. The plates were then held at either 37 °C for 4 h or 4 °C for 2 h prior to fixation with 4% p-formaldehyde. Staining was performed by incubating the cells in a 0.5ug/mL solution of DAPI in PBS for 30 min on a plate shaker. After the excess stain was removed with 3 washes of 150 μl PBS, pictures were taken on 20 × magnification with a fluorescence microscope in both the DAPI (excitation wavelength: 377, emission wavelength: 447) and Texas Red (excitation wavelength: 586, emission wavelength: 647) channels. Analysis of the images was performed in Gen5 v3.08. For all images, nuclei were identified by finding objects in the DAPI channel with an intensity greater than 10,000 between 5 μm and 30 μm in diameter. For cells held at 37 °C, diI-oxLDL uptake was quantified by finding the integral intensity of a continuous area of red signal with an intensity greater than 8,000 propagated out from the nucleus of each cell. For cells held at 4 °C, diI-oxLDL binding was quantified by finding the integral intensity of all areas of red signal with an intensity greater than 10,000 around each nucleus.

### Measurement of neutral lipids

BMMs were grown to confluence in 48 well plates in RPMI 1640 media supplemented with 10% FBS, 1x Antibiotic-Antimycotic, and 15% L929 conditioned media. After each treatment, Cholesterol/Cholesterol Ester-Glo™ Assay (Promega) was used to measure total cholesterol and free cholesterol. Serial dilutions were prepared from a cholesterol standard supplied in the kit. Total cholesterol and free cholesterol concentrations were calculated from the luminescence values of the lysates with and without cholesterol esterase respectively. The amounts of lipids were normalized by protein concentration. Free cholesterol concentrations were then subtracted from total cholesterol concentrations to find the concentration of cholesterol ester.

### Immunoblotting

BMMs were cultured in 6 well culture plates at the density of 5 ~ 8 × 10^5^ cells per well for 24 h. After treating cells as indicated in figure legends, cells were rinsed with cold 1xPBS, and the protein was extracted in RIPA buffer. Twenty five microgram of protein was loaded per lane and immunoblot was performed with each antibodies shown in the figures.

### IL-1β secretion

BMMs were cultured in a white 96 well culture plate at the density of 14,000 cells per well for 24 h. Cells were treated with oxLDL (25μg/ml) or oxLDL (25μg/ml) plus LPS (10 ng/ml) for 24 h. Then verteporfin was treated at 2.5μM final concentration for 24 h. The secreted IL-1β to media was measured using Lumit™ IL-1β mouse immunoassay kit (Promega).

### Capase-1 activity

BMMs were cultured in 6 well culture plates for 1 day, and 25 μg/ml of oxLDL was treated for 24 h with or without 10 ng/ml of LPS. After refreshing media, cells were treated with verteporfin at 2.5 μM for 24 h. Cells were treated with nigericin (5 μM) for 30 min. Culture supernatant was collected, and cells were lysed in RIPA buffer to extract protein and immunoblotting was performed as described above. Fifty microliter of supernatant was used to measure capase-1 activity using Capase-Glo1 Inflammasome Assay (Promega).

### Cell apoptosis

BMMs were cultured in a black 96 well culture plate at the density of 10,000 cells per well for 24 h. Cells were treated with oxLDL (25 μg/ml) for 24 h and then treated with and without verteporfin (2.5 μM) for additional 24 h. As a control, staurosporine (2.5 μM) was treated for 24 h. After washing, cells were fixed with 4% PFA, and cell death was detected using In Situ cell death detection kit, TMR red (Roche) according to the manufacturer’s instruction.

### Statistical analysis

All values are expressed as mean ± SD. A two-tailed student *t* test using Graph Pad Prism 9.51 (La Jolla, CA) was used to determine statistical significance between control and treated groups. Values of *P* < 0.05 were considered as significant.

### Supplementary Information

Supplementary Figures.

## Data Availability

Results of all compounds in the library are available upon request to the corresponding author (gooy@amc.edu).
