# Supplementary material for: Drug screen identifies verteporfin as a regulator of lipid metabolism in macrophage foam cells
Source: Sci Rep. 2023 Nov 9;13:19588. doi: 10.1038/s41598-023-46467-4 (PMC10638409; doi:10.1038/s41598-023-46467-4)

Supplementary Figure 1.

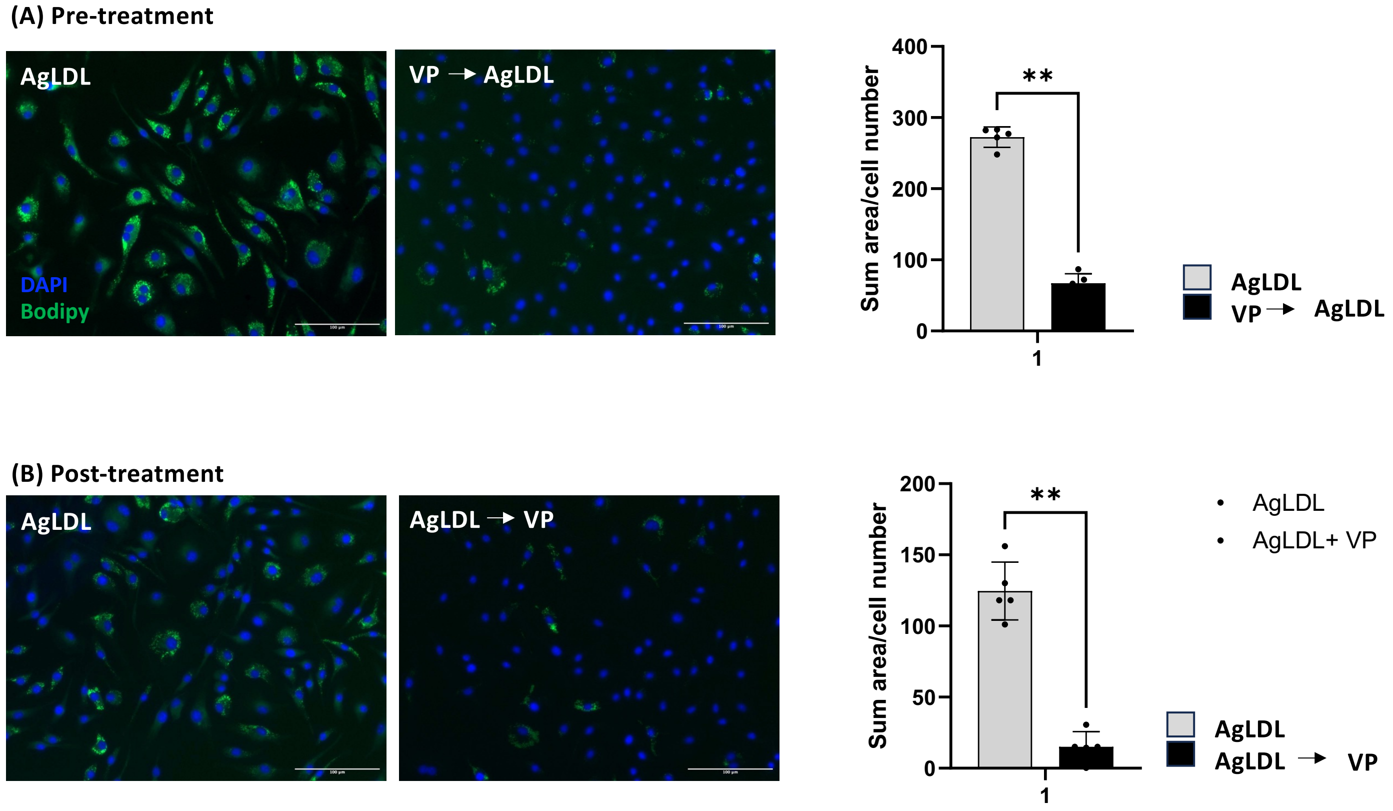

Supplementary Figure 1. Verteporfin on AgLDL-induced foam cell formation and regression.

VP (10ug/ml) was treated 30min before AgLDL (25ug/ml) loading **(A)** or was added after 24hr of AgLDL (25ug/ml) treatment **(B).** Representative images of foam cells (left) and measurement of lipid contents (right). Scale bar; 100μm. *P* values are by Student's t-test for paired samples. N=5, **; $p<0.005$.

Supplementary Figure 2.

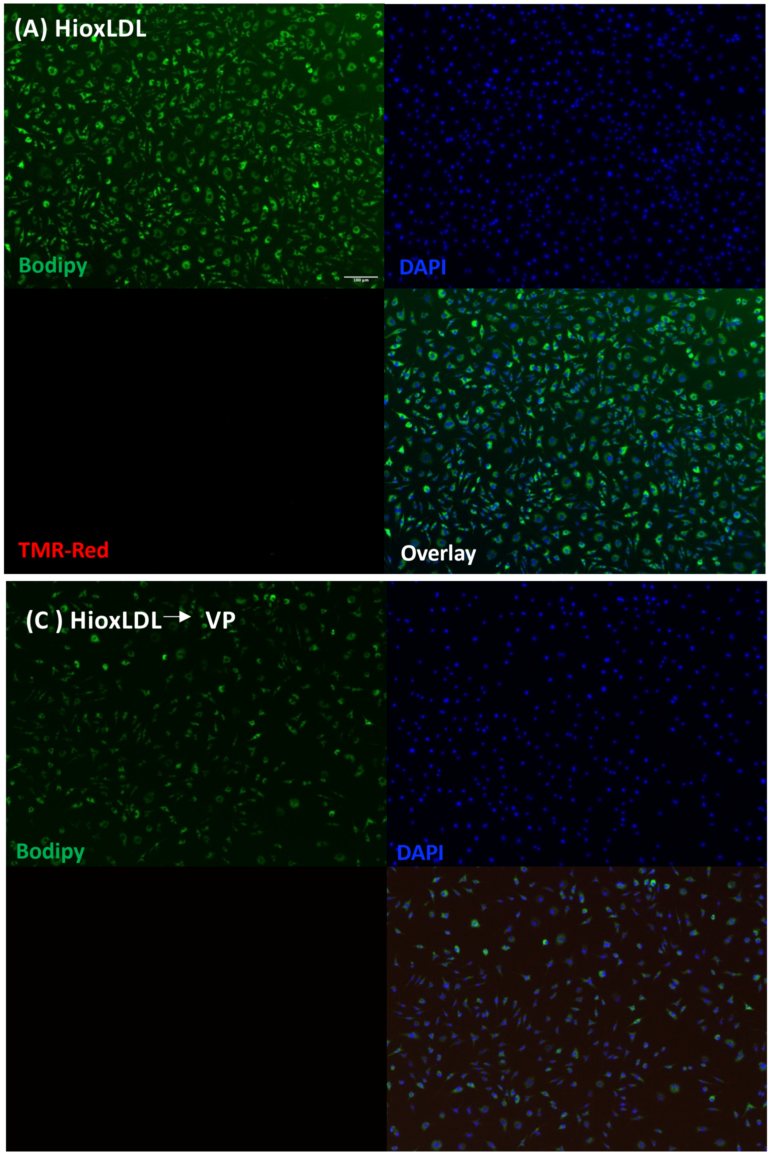

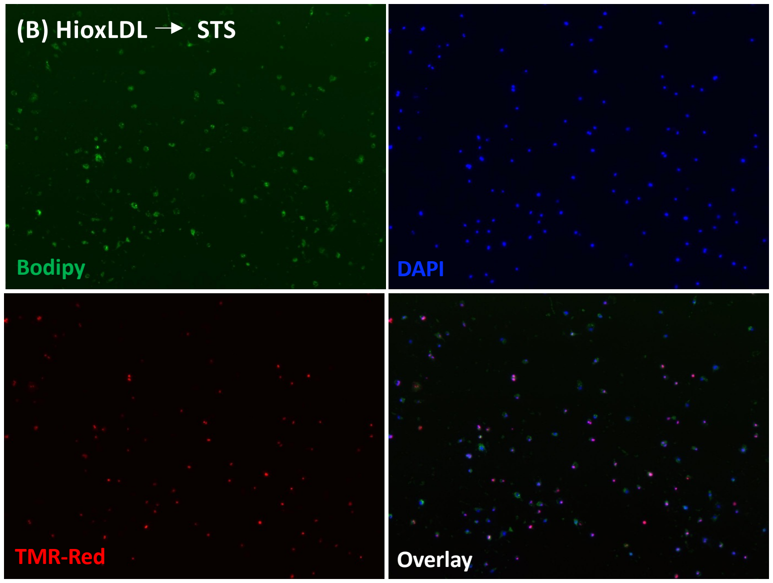

Supplementary Figure 2. Detection of cell apoptosis. BMM cells were treated with oxLDL for 24hrs. Then vehicle **(A)**, staurosporine **(B)**, or verteporfin **(C)** were added for additional 24hr. Bodipy 493/503 (green) for LDs, DAPI (blue) for nucleus, and TMR-Red (red) for detection of apoptotic cells were used. Scale bar 100μm.

Supplementary Figure 3. WB images

Figure. 6B

ABCA1 (>250 kDa)

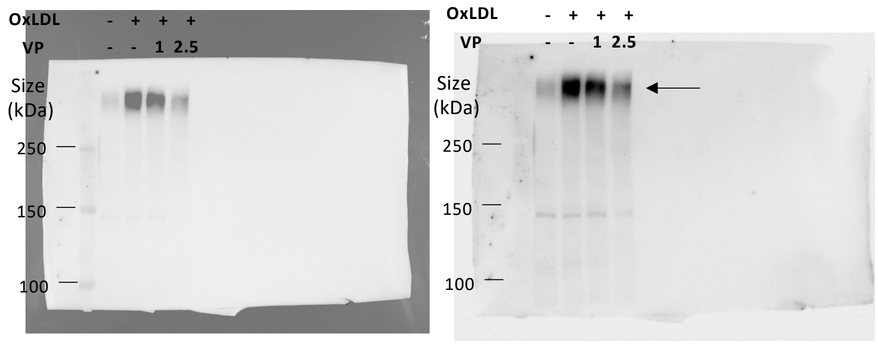

CD36 (110 kDa)

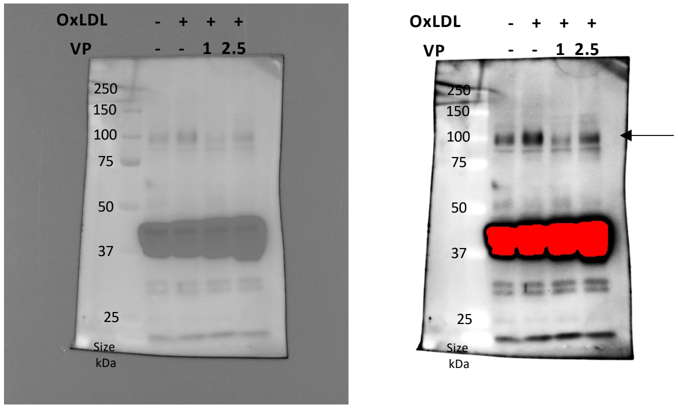

PLIN2 (48 kDa)

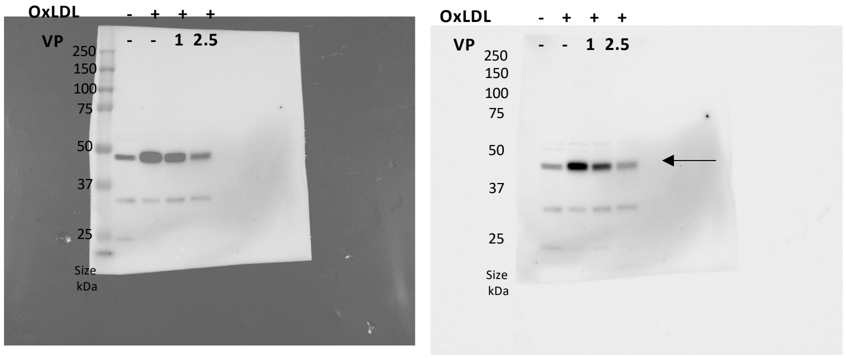

ACTIN (42 kDa)

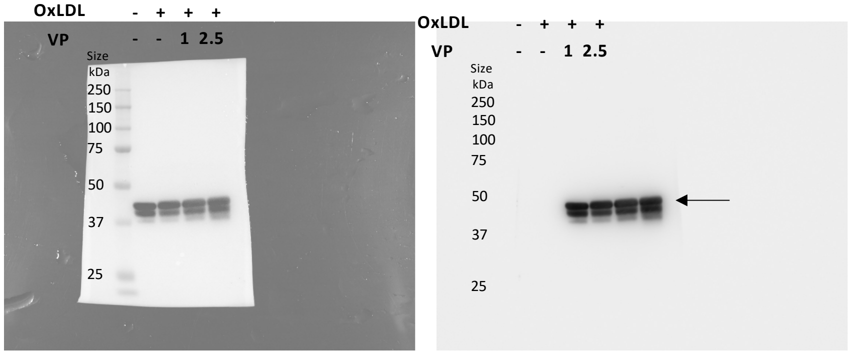

Supplementary Figure 3. WB images (continued)

Figure. 7B

CD36 (110 kDa)

VP

- - 1 2.5 5 Rot

OxLDL - + + + + +

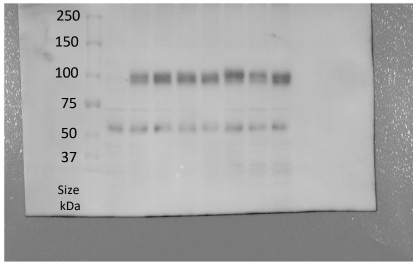

VP

- - 1 2.5 5 Rot

OxLDL - + + + + +

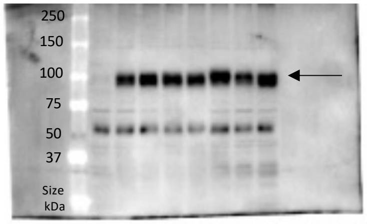

PLIN2 (48 kDa)

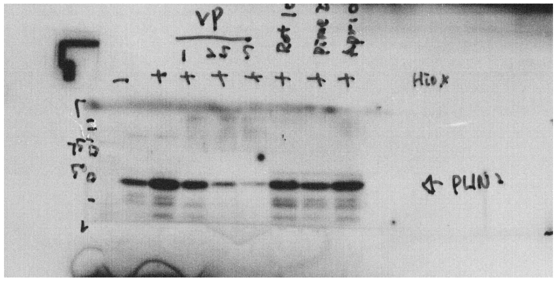

ACTIN (42 kDa)

VP

- - 1 2.5 5 Rot

Size kDa - + + + + +

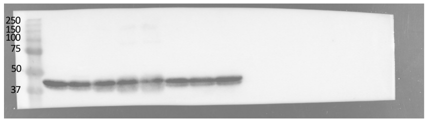

VP

- - 1 2.5 5 Rot

Size kDa - + + + + +

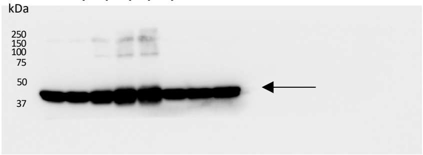

Supplementary Figure 3. WB images (continued)

Figure. 7F

NLRP3 (118 kDa)

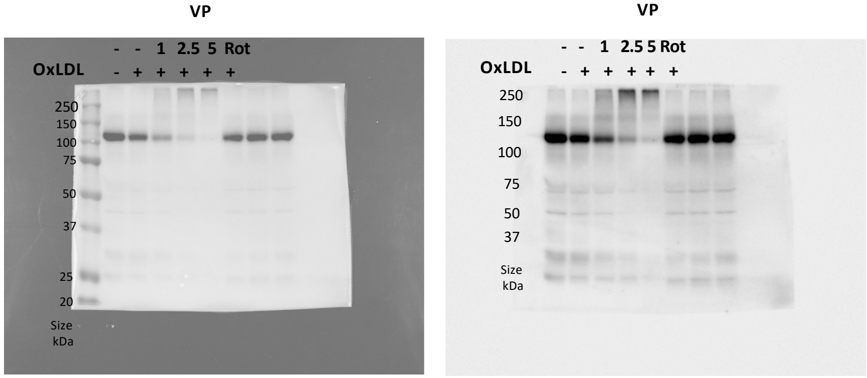

ABCA1 (>250 kDa)

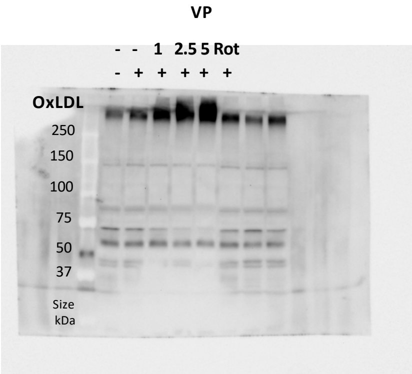

ASC (22 kDa)

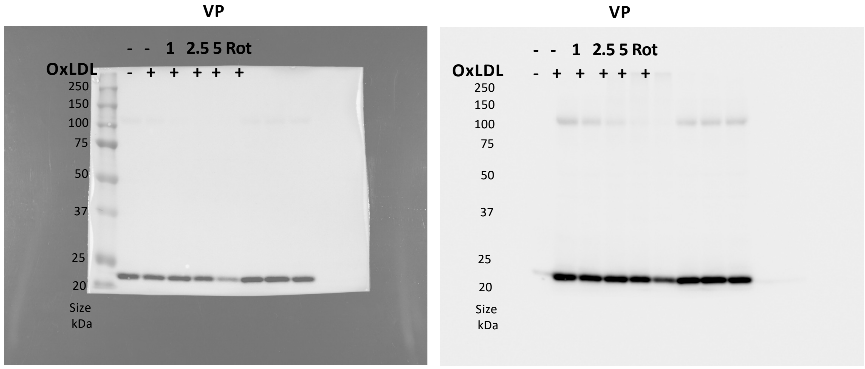

ACTIN (42 kDa)

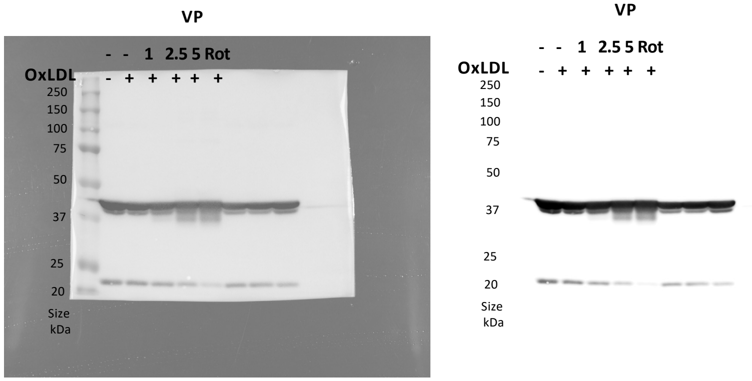

Supplement: Supplementary file 1 — Supplementary Figures. [file 41598_2023_46467_MOESM1_ESM.pdf]
